# Polyglutamine-Rich Suppressors of Huntingtin Toxicity Act Upstream of Hsp70 and Sti1 in Spatial Quality Control of Amyloid-Like Proteins

**DOI:** 10.1371/journal.pone.0095914

**Published:** 2014-05-14

**Authors:** Katie J. Wolfe, Hong Yu Ren, Philipp Trepte, Douglas M. Cyr

**Affiliations:** 1 Department of Cell Biology and Physiology, University of North Carolina, Chapel Hill, North Carolina, United States of America; 2 Neuroproteomics, Max Delbrueck Center for Molecular Medicine, Berlin, Germany; Boston University Medical School, United States of America

## Abstract

Protein conformational maladies such as Huntington Disease are characterized by accumulation of intracellular and extracellular protein inclusions containing amyloid-like proteins. There is an inverse correlation between proteotoxicity and aggregation, so facilitated protein aggregation appears cytoprotective. To define mechanisms for protective protein aggregation, a screen for suppressors of nuclear huntingtin (Htt103Q) toxicity was conducted. Nuclear Htt103Q is highly toxic and less aggregation prone than its cytosolic form, so we identified suppressors of cytotoxicity caused by Htt103Q tagged with a nuclear localization signal (NLS). High copy suppressors of Htt103Q-NLS toxicity include the polyQ-domain containing proteins Nab3, Pop2, and Cbk1, and each suppresses Htt toxicity via a different mechanism. Htt103Q-NLS appears to inactivate the essential functions of Nab3 in RNA processing in the nucleus. Function of Pop2 and Cbk1 is not impaired by nuclear Htt103Q, as their respective polyQ-rich domains are sufficient to suppress Htt103Q toxicity. Pop2 is a subunit of an RNA processing complex and is localized throughout the cytoplasm. Expression of just the Pop2 polyQ domain and an adjacent proline-rich stretch is sufficient to suppress Htt103Q toxicity. The proline-rich domain in Pop2 resembles an aggresome targeting signal, so Pop2 may act in trans to positively impact spatial quality control of Htt103Q. Cbk1 accumulates in discrete perinuclear foci and overexpression of the Cbk1 polyQ domain concentrates diffuse Htt103Q into these foci, which correlates with suppression of Htt toxicity. Protective action of Pop2 and Cbk1 in spatial quality control is dependent upon the Hsp70 co-chaperone Sti1, which packages amyloid-like proteins into benign foci. Protein:protein interactions between Htt103Q and its intracellular neighbors lead to toxic and protective outcomes. A subset of polyQ-rich proteins buffer amyloid toxicity by funneling toxic aggregation intermediates to the Hsp70/Sti1 system for spatial organization into benign species.

## Introduction

A group of human maladies termed conformational disorders (CD) result from misfolding and aggregation of different underlying disease proteins [1]. Intracellular and extracellular inclusions found in tissue of affected individuals contain amyloid-like proteins that are enriched in beta-sheet structure, detergent insoluble, and bind amyloid indicator dyes [1]–[4]. Conversion of native proteins to an amyloid-like state occurs spontaneously and is often seeded through interactions with other proteins that are in an amyloid-like state [5]. Templated protein species self-associate and form a variety of assemblies that include small oligomers, amyloid-like fibers and plaques [6]–[8]. Whether the large amyloid-like assemblies detected in disease states are themselves causative or a protective cellular defense mechanism against an unknown toxic species is a topic of debate [3]. The harmful species in CD may be small oligomeric intermediates of the templated aggregation pathway while accumulation of larger benign assemblies could be a by-product of such a pathway or a productive defense mechanism [9]–[16]. Therefore, protection against proteotoxicity in CD involves preventing entry of native proteins into the amyloid-like assembly pathway or, once entry has already occurred, driving the formation of a benign aggregated species. In both cases, protein quality control (PQC) factors such as the molecular chaperone Hsp70 act to prevent accumulation of toxic protein species.

To study basic cellular mechanisms for suppression of proteotoxicity caused by proteins that contain expanded polyglutamine (polyQ)-rich repeats, we employ yeast that express huntingtin containing an expanded polyQ domain (Htt103Q) [6], [17]. Onset of Huntington Disease (HD) in humans occurs upon protease cleavage of the full length Htt protein into N-terminal exon 1 Htt fragments followed by translocation into the nucleus where the fragments accumulate in inclusions [18], [19]. Aggregates of Htt103Q have biochemical characteristics of amyloid, so they are considered to be amyloid-like. Investigators express fragments of Htt103Q that correspond to exon 1 in yeast and other model systems to study basic mechanisms for suppression of proteotoxicty encountered in CD [6], [13], [20].

Both cytoplasmic and intranuclear Htt inclusions are associated with HD [21]–[23], but toxicity and behavioral phenotypes are more severe when Htt accumulates in the nucleus [17], [24], [25]. The nucleus contains a large number of glutamine rich proteins that are involved in DNA and RNA metabolism so entrance of Htt103Q into the nucleus could directly lead to their inactivation [26]–[29]. In the cytosol, toxic species of Htt induce ER stress, possibly by sequestration of p97/VCP [30]. While a clear mechanistic understanding of nuclear versus cytosolic polyQ toxicity remains to be discovered, several factors have been implicated in this process. Molecular chaperones prevent formation or detoxify toxic protein species [31]–[33], and the relative concentration of such proteostatic factors in the cytosol and nucleus are different. This creates unique environments that have different buffering capacities for toxic protein species and spatial quality control factors, and the nuclear environment may not be as well-equipped as the cytosol to detoxify Htt. Indeed, soluble species of Htt103Q are more prominent in the nucleus and nuclear Htt appears more toxic than cytosolic forms [17].

In yeast models for polyQ disease, onset of Htt103Q toxicity is dependent on the conversion of benign Htt103Q to a toxic form through physical interaction with amyloid-like forms of the glutamine/asparagine-rich yeast prion protein [*RNQ+*] [6], [34]. Interaction of Htt103Q and [*RNQ+*] prion results in the conversion of benign soluble Htt103Q to a poorly defined toxic conformer whose formation is associated with accumulation of Htt103Q as a soluble species and detergent insoluble oligomers [6], [34]. Htt103Q toxicity in yeast is also sensitive to the presence of a proline-rich domain that flanks the polyQ domain [35], [36]. Htt103Q-Pro accumulates in large perinuclear aggregates in a process facilitated by the chaperone p97 and is non-toxic [35]. Interestingly, deletion of the proline-rich domain leads to cytotoxicity and decreases the efficiency of Htt-103Q aggregation, which correlates with Htt103Q accumulation in a mixture of small foci and unassembled species [17], [36]. Therefore, inefficiencies in packaging of Htt103Q into intracellular assemblies correlate with the onset of Htt toxicity in yeast. These data suggest that spatial quality control mechanisms play an important role in suppression of proteotoxicity, yet mechanisms for packaging of polyQ containing proteins into benign assemblies are not well understood.

The Hsp70 chaperone system acts to suppress proteotoxicity via multiple mechanisms [37]–[39]. The Type II Hsp40 Sis1 and Hsp70 cooperate with different spatial quality control factors to direct misfolded proteins to specific protein quality control (PQC) machinery and subcellular deposition sites [40]–[42]. Hsp70 and Sis1 also cooperate with the ubiquitin ligases Ubr1 and San1 to degrade short lived cytosolic proteins [42] with misfolded proteins being targeted to a juxtanuclear quality control compartment (JUNQ) when the ubiquitin proteasome system is saturated [40]. Sis1 levels also influence whether [*RNQ+*] prions are predominantly localized in the cytosol or nucleus, with elevation of Sis1 shifting [*RNQ+*] prions from the cytosol to the nucleus [17]. Interestingly, when [*RNQ+*] prions are localized in the nucleus, Htt103Q also accumulates in the nucleus, and is more toxic because the nucleus has a reduced capacity to package it into benign assemblies [17]. Sti1 is a TPR-repeat containing Hsp70 co-chaperone and cooperates with Sis1 to sequester Htt103Q as well as [*RNQ+*] prions into benign perinuclear foci that appear to be distinct from the JUNQ [43]. Chaperone assisted protein aggregation plays an important role in suppression of proteotoxicity, so it is important to understand this process in more detail.

In order to identify cellular mechanisms for packaging of proteotoxic protein species into benign states, we carried out a high copy toxicity suppressor screen utilizing Htt103Q-NLS as the substrate. The study of nuclear Htt103Q toxicity, which is more toxic yet more detergent soluble, allowed us to identify suppressors of proteotoxicity that impact spatial organization and aggregation of amyloid-like Htt species. Although numerous other studies have screened for factors affecting polyQ toxicity or aggregation [6], [44]–[53], the use of nuclear Htt103Q permitted identification of novel cellular factors that facilitate spatial PQC.

Herein we describe three Htt toxicity suppressors, each of which contain a polyQ-rich region, and act via different biochemical mechanisms. Nab3 is an essential protein that is inactivated by Htt103Q-NLS. Overexpression of Nab3 has no effect upon Htt aggregation and restores Nab3 function to alleviate toxicity. In contrast, Pop2 and Cbk1 both alter spatial organization of Htt aggregates while suppressing toxicity. The polyQ-rich regions of Pop2 and Cbk1 are necessary and sufficient to suppress toxicity, and do so by concentrating small Htt103Q foci into larger benign assemblies. Changes in the spatial organization of Htt103Q foci caused by Pop2 and Cbk1 are dependent upon the Hsp70 co-chaperone Sti1 [43]. PolyQ proteotoxicity can be suppressed through interactions of Htt103Q with non-toxic polyQ-rich proteins that funnel toxic Htt species into the Sti1/Hsp70 dependent spatial quality control pathway.

## Results

### Identification of High Copy Suppressors of Htt103Q-NLS Toxicity in Yeast

**Figure 1:**
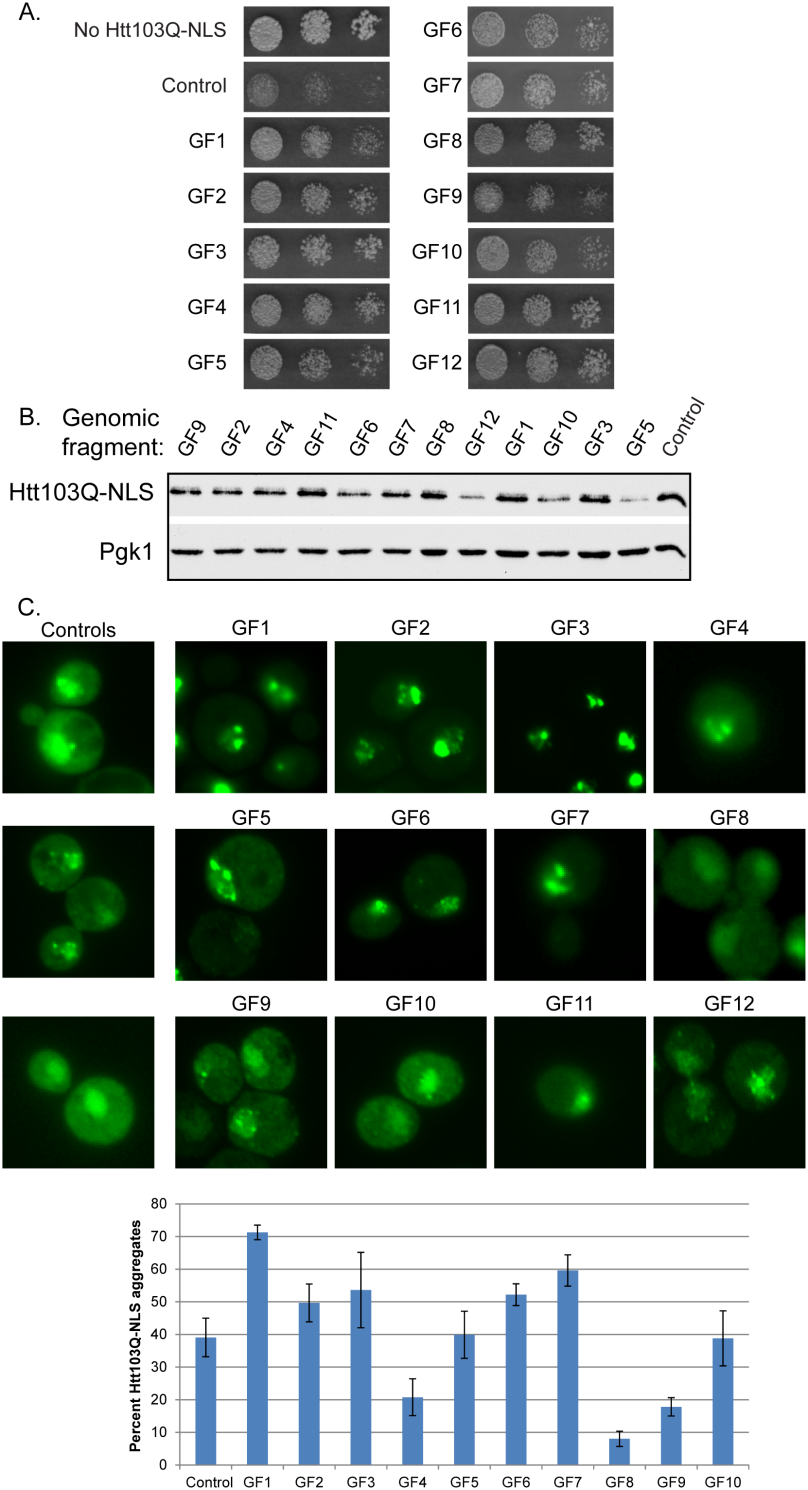
Genomic fragments from high copy screen suppress Htt103Q-NLS toxicity. (A) Genomic fragments (GF) 1 through 12 suppress Htt103Q-NLS toxicity as monitored by growth assay plated in 5-fold dilutions on galactose containing media. (B) Impact of GF1-12 upon Htt103Q-NLS expression levels as monitored by Western blot. (C) Impact of GF1-12 upon aggregation of Htt103Q-NLS as monitored by fluorescence microscopy. Bar graph indicates percent of cells with Htt103Q-NLS found in intranuclear foci. Each bar indicates the average ± sd of at least 100 cells counted in at least 3 different experiments. Cultures for Western blotting and microscopy were obtained by inducing Htt103Q-NLS with 2% galactose for 4–5 h.

In order to understand mechanisms for protective aggregation and spatial organization of toxic polyQ species, we screened a yeast high copy expression library for suppressors of growth defects caused by Htt103Q-NLS [17]. This form of Htt103Q contains an N-terminal FLAG tag, the first 17 amino acids encoded by HTT exon 1, a polyQ stretch of 103 amino acids, a C terminal GFP tag, and the SV40 nuclear localization signal (NLS). Notably Htt103Q-NLS lacks the proline-rich region that flanks the polyQ domain in full length Htt, so it has reduced aggregation propensity and is therefore toxic in strains that contain [*RNQ+*] prions [6]–[8], [35], [36]. The gene for Htt103Q-NLS was integrated into strain W303α under a galactose inducible promoter, and a growth defect is observed when this strain is plated on solid media containing galactose (Figure 1A). This strain was transformed with a yeast multicopy expression library [22], and colonies that were visible after 3 days of growth on galactose were isolated. Following plasmid rescue and sequencing of 23 plasmid dependent suppressors, it was evident that several hits contained overlapping regions of DNA suggesting that the screen neared saturation (Table 1 and Figure 1A).

**Table 1:** Genomic fragments that suppress Htt103Q-NLS toxicity.

Gen. fragment#	gene	biological process*	polyQ rich	Null	Independent
1	STI1	protein folding, Hsp90	No	viable	Yes
	CIN5	RNA pol II transcription factor activity	No	viable	No
2	POP2	deadenylation, CCR4/NOT complex	Yes	viable	Yes
	BRE5	Ubiquitin protease cofactor, deubiquitination	No	viable	No
3	CBK1	Ser/Thr protein kinase	Yes	viable**	Yes
	YGP1	Response to nutrient/stress	No	viable	No
	ASI2	ubiquitin-dependent protein catabolic process	No	viable	No
	PGA1	mannosyltransferase activity	No	inviable	No
4	GUP2	glycerol transport	No	viable	No
	COA2	Cytochrome oxidase assembly factor	No	viable	No
	NAB3	pol II regulation	Yes	inviable	Yes
5	ZDS2	transcriptional silencing	No	viable	No
	YML108W	unknown	No	viable	No
	PML39	mRNA export from nucleus	No	viable	No
	URA5	pyrimidine biosynthesis	No	viable	No
	SEC65	protein targeting to ER	No	inviable	No
	MDM1	mito. Inheritance, nuc migration	No	viable	No
	NUP188	import and export from nucleus	Yes	viable	No
6	SCD5	actin cytoskelton organization	Yes	inviable	No
	PDR10	ABC transporter	No	viable	No
7	SUL2	sulfate transport	No	viable	No
	NYV1	vesicle fusion	No	viable	No
	GIS3	unknown	No	viable	No
	IOC2	chromatin remodeling	Yes	viable	No
8	BUD22	bud site selection	No	viable	No
	ERG5	ergosterol biosynthetic process	No	viable	No
	SOK2	pseudohyphal growth, transcription factor	Yes	viable	No
	SPO20	ascospore-type prospore formation	No	viable	No
9	YHR122w	unknown, required for chromatid cohesion	No	inviable	No
	EPT1	phosphatidylethanolamine synthesis	No	viable	No
	NDT80	Transcription factor	No	viable	No
	YHR126c	unknown	No	viable	No
	YHR127w	mitotic spindle elongation	No	viable	Yes
	FUR1	pyrimidine salvage	No	viable	No
	ARP1	nuclear migration	No	viable	No
10	SEC3	transport, vesicle fusion	No	inviable	No
	NTF2	protein import into nucleus	No	inviable	No
	YER010C	unknown	No	viable	No
	TIR1	response to stress	No	viable	No
11	SOK1	cAMP mediated signaling	No	viable	nd
	TRP1	tryptophan biosynthesis	No	viable	nd
	GAL3	galactose metabolism	No	viable	nd
	SNQ2	ABC transporter, response to drug	No	viable	nd
12	MIG1	transcription factor, glucose repression	No	viable	nd

nd- not determined.

*from Saccharomyces Genome Database.

**viability attributed to ssd1-d allele in the W303 background.

Twelve genomic DNA fragments (GF), designated GF1 to 12, which alleviated Htt103Q-NLS toxicity were isolated (Table 1 and Figure 1A). Then each of these GFs was analyzed for how it impacted Htt103Q-NLS expression and aggregation. GF 5, 6, 10, and 12 decreased steady state protein levels of Htt103Q-NLS (Figure 1B), which was likely the reason for suppression of Htt103Q-NLS toxicity. GF 11 contained an ORF encoding proteins involved in sugar metabolism. GF 5, 6, 10, 11, and 12 do not appear to alter spatial organization of Htt103Q, and were eliminated from further validation.

GF 1–3, 6 and 7 had an impact upon the organization of intranuclear Htt103Q-NLS as monitored both by intensity of fluorescent foci and number of cells containing nuclear foci (Figure 1C), so they were studied in detail. For reference purposes, examples of control cells with diffuse Htt103Q-NLS, as well as intranuclear foci, are shown (Figure 1C). The GFs of interest each contained a fragment of genomic DNA approximately 5–10 Kb long that included 1–7 ORFs (Table 1). In order to determine the protein responsible for suppressing Htt103Q-NLS toxicity, individual ORFs were subcloned and expressed in yeast under control of its endogenous promoter. Table 1 shows the individual proteins encoded by OFs in different GFs that suppressed Htt103Q-NLS toxicity. Notably, 3 of the 7 GFs containing an ORF that encoded a protein with a polyQ-rich region could independently suppress Htt toxicity (Table 1). These polyQ containing genes include POP2, CBK1, and NAB3. Another ORF, lacking a polyQ domain, found to be an independent suppressor of Htt103Q-NLS toxicity was the Hsp70 co-chaperone STI1. The suppressive action of Sti1 on Htt toxicity was characterized in a separate study [43]. It acts with Hsp70 in spatial quality control of amyloid-like proteins to suppress Htt103Q toxicity by promoting accumulation of amyloid-like material in benign perinuclear foci [43]. Herein, we investigated the mechanism by which the polyQ-rich proteins Pop2, Cbk1, and Nab3 suppress toxicity of Htt103Q.

### PolyQ-rich Proteins Suppress Htt103Q-NLS and Htt103Q Toxicity

**Figure 2:**
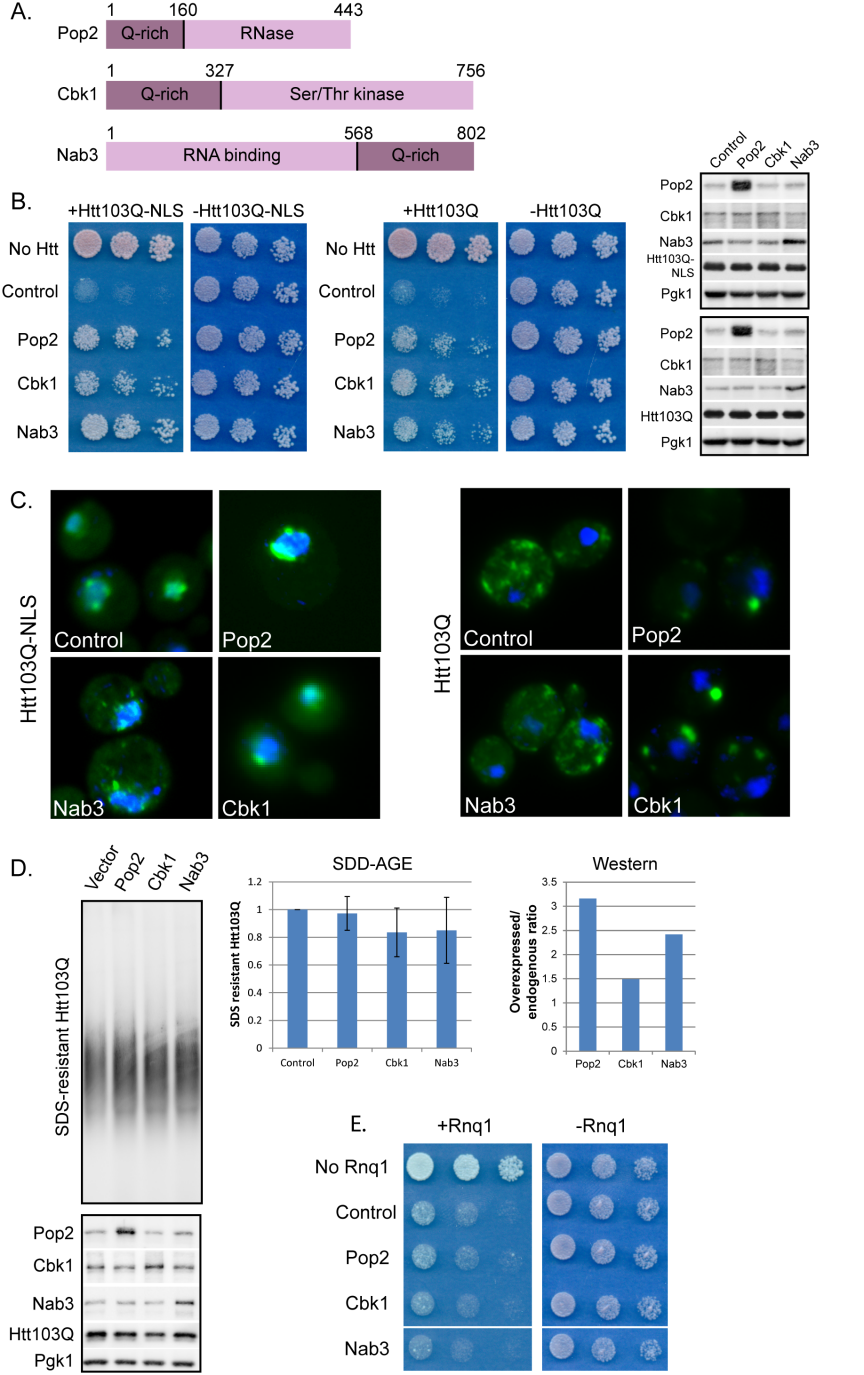
PolyQ-rich proteins suppress Htt103Q-NLS and Htt103Q toxicity. (A) Diagram of polyQ-rich screen hits. Numbers represent amino acids. N- or C-terminal polyQ-rich regions and functional domains are indicated. Further information is given in . (B) Pop2, Cbk1, and Nab3 suppress growth defect associated with Htt103Q-NLS and Htt103Q. Growth assays were plated in 5-fold dilutions on glucose (−Htt) or galactose (+Htt). Western blot indicates Htt expression levels at 4 h galactose induction with indicated proteins co-expressed. (C) Pop2 and Cbk1 but not Nab3 alter Htt103Q-NLS and Htt103Q aggregation as monitored by fluorescence microscopy. Nuclei were visualized with DAPI staining. (D) Pop2, Cbk1, and Nab3 do not alter SDS-resistant Htt103Q aggregation as monitored by SDD-AGE and Western blot analysis. Graphs indicate quantitation of SDS-resistant Htt103Q (N = 3, SE indicated) and ratio of overexpressed Pop2, Cbk1, or Nab3 over endogenous. (E)Toxicity associated with Rnq1 over-expression is unaffected by screen hits. Growth assays were plated in 5-fold dilutions on glucose (−Rnq1) or galactose (+Rnq1). Endogenous Rnq1 is present in a [RNQ+] prion state in this experiment. Pop2, Cbk1, and Nab3 were expressed from high copy plasmids under control of their endogenous promoters in each experiment. Htt103Q was induced with 2% galactose for 4 h.

Pop2 and Cbk1 contain polyQ-rich regions N-terminal to the functional domain of each protein, while Nab3 contains a polyQ-rich region located C-terminal to the functional domain (Figure 2A). Each of these “polyQ-rich” regions contains several stretches of Q repeats of 6 or more residues (Figure S1A and S1B). While amino acids in between these polyQ tracts are enriched in hydrophobic residues, there is little sequence similarity between the polyQ-rich regions of these 3 proteins besides the polyQ tracts themselves (Figure S1C). These polyQ-rich domains are predicted to have a relatively high propensity for being disordered [54] suggesting they are conformationally dynamic. Conformational switching is a characteristic of prion proteins which can be folded normally or adopt a self-propagating amyloid-like conformation. Pop2, Cbk1, and Nab3 were all identified in a bioinformatics screen for putative prion domain containing proteins [55]. The criteria for determining prion domains were based upon an algorithim that searched for sequences similar to the prion domains of the yeast prions Sup35, Ure2, and Rnq1. Based upon the prion domain forming ranking system for the top 100 hits, Cbk1, Nab3, and Pop2 fell at positions 6, 41, and 51, respectively [55]. When expressed in yeast, the prion domains of Cbk1 and Pop2 formed SDS-resistant oligomers, while Nab3 did not [55]. Thus, Cbk1 and Pop2, but not Nab3, contain a polyQ-rich region that has an intrinsic propensity to assume an amyloid-like conformation.

Overexpression of Pop2, Cbk1, and Nab3 each partially suppressed toxicity of both Htt103Q-NLS and a similar construct lacking the NLS (Htt103Q), which aggregates in the cytosol (Figure 2B). Reduced toxicity was not due to altered levels of the Htt proteins (Figure 2B). Pop2 and Cbk1, but not Nab3, overexpression suppressed Htt103Q-NLS toxicity in a manner that correlated with changes in spatial organization of Htt103Q (Figure 2C). One of the features of toxic forms of Htt is that they exhibit a characteristic of amyloid-like proteins and form SDS-resistant protein oligomers [56], [57]. Therefore, we asked if total amounts of SDS-resistant oligomers were altered by Pop2, Cbk1, or Nab3. Under expression conditions where the over-expressed polyQ-rich protein could suppress Htt103Q toxicity, there was little change in SDS-resistant Htt103Q as measured by SDD-AGE (Figure 2D). Htt103Q toxicity and aggregation in yeast depends upon the length of the polyglutamine expansion within the HTT gene and the templated status of the prion protein Rnq1 [6], [34]. Pop2, Cbk1, or Nab3 have no impact on the spatial organization of benign, diffuse Htt25Q in [*RNQ+*] strains. The effect of these proteins on non-aggregated Htt103Q in a [rnq*−*] yeast strain was not examined as Htt103Q is not toxic in this strain. Since Cbk1 and Pop2 cause cytosolic speckled Htt103Q-GFP to condense in to one or two perinuclear foci, but do not impact aggregation, they appear to suppress proteotoxicity via modulating spatial organization of Htt103Q.

To evaluate the specificity of these suppressors, whether they alleviated the growth defect associated with Rnq1 overexpression was determined. These polyQ-rich proteins do not appear to act on [*RNQ+*] prions as they were unable to suppress toxicity associated with Rnq1 overexpression (Figure 2E). When Rnq1 is in the [*rnq−*] non-prion conformation, Htt103Q is no longer toxic, so the inability of the suppressor to influence Rnq1 toxicity indicates that they are not changing spatial quality control of [*RNQ+*] prions. This interpretation is supported with experimental data showing that Pop2 expression does not change the organization of Rnq1-GFP foci (Figure S2A). Likewise, the polyQ-rich suppressors did not co-localize with Rnq1 aggregates (Figure S2A). Thus, Pop2, Cbk1, and Nab3 over-expression modulates toxicity of Htt103Q without having a detectable impact upon spatial organization of [*RNQ+*] prions.

As Cbk1 and Pop2 alter Htt103Q aggregation and Nab3 does not, these proteins appear to fall into different classes of polyQ-rich proteins that suppress Htt103Q toxicity. Additionally, although the polyQ-rich proteins identified in our screen suppress polyQ expanded Htt toxicity, other polyQ-rich proteins actually enhance toxicity [58]. Some toxicity enhancers also ranked within the top100 in the above mentioned bioinformatics screen for proteins containing prion domains [55]. These data implicate populations of different polyQ-rich proteins in the cellular environment as having a multifaceted role in impacting proteotoxicity of a polyQ disease related protein. Therefore, we sought to distinguish the different mechanisms of toxicity suppression by the polyQ proteins under study.

### Functional Nab3 is Required for Suppression of Htt Toxicity

**Figure 3:**
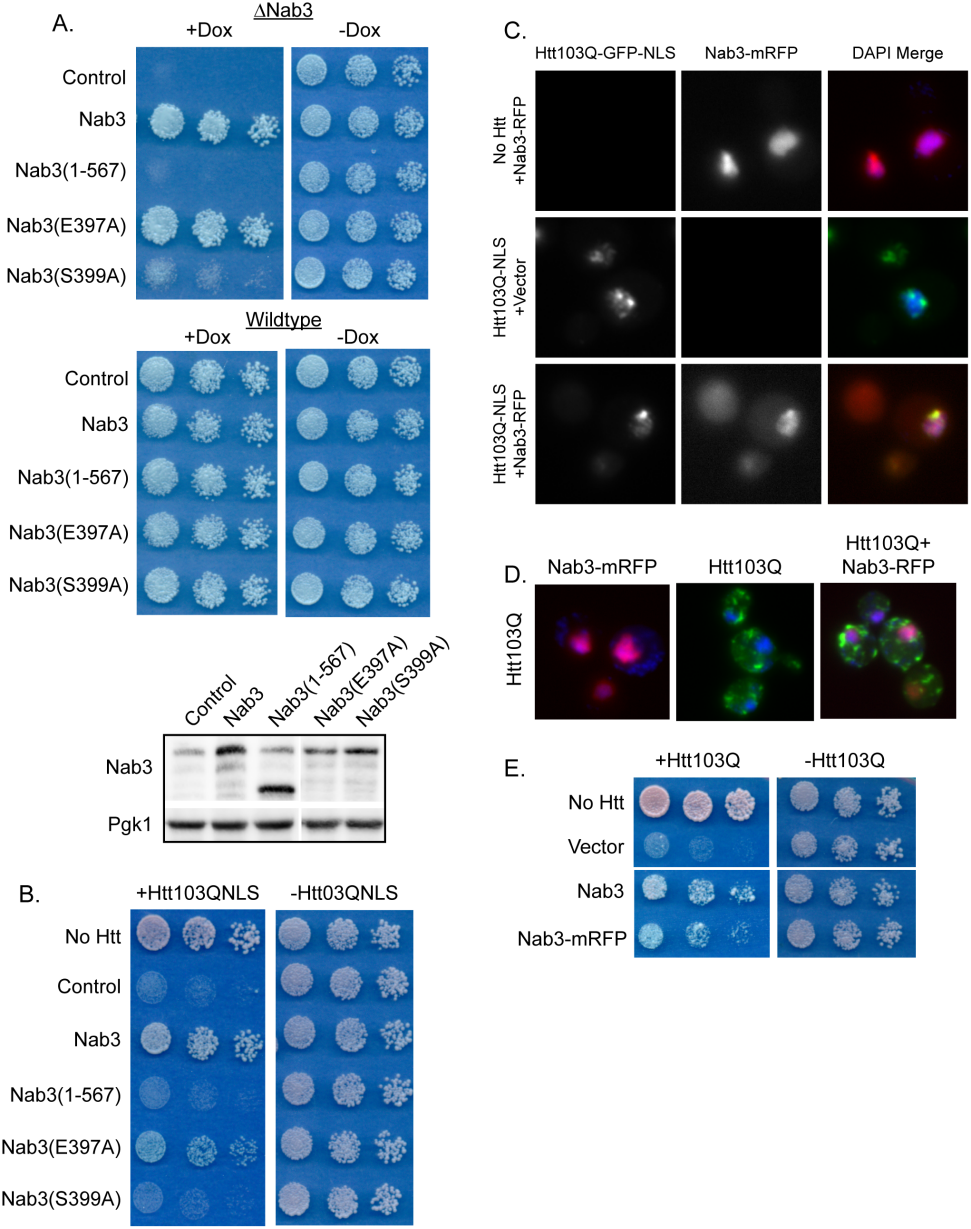
Overexpression of Nab3 must be functional for Htt103Q-NLS toxicity suppression. (A) Nab3 requires both the polyQ-rich region and a functional RNA recognition motif to complement Nab3 deletion. Growth assays were plated in 5-fold dilutions on glucose plates in the presence or absence of 10 ug/mL doxycycline which inhibits Nab3 expression in this strain. Western blots indicate expression levels of Nab3 constructs, each of which are under control of the endogenous Nab3 promoter. (B) Over-expression of non-functional Nab3 does not suppress Htt103Q-NLS toxicity as monitored by growth assays plated in 5-fold dilutions. Nab3(1–567) lacks the C-terminal polyQ-rich region of the protein. (C) Nab3-mRFP co-localizes with Htt103Q-NLS in the nucleus and in intranuclear foci as monitored by fluorescence microscopy. (D) Nab3-mRFP does not co-localize with Htt103Q. Nuclei were visualized with DAPI staining. (E) Nab3-mRFP suppresses toxicity of Htt103Q.

Nab3 is an essential nuclear protein that functions in the Nrd1 complex as an RNA binding protein through its RNA recognition motif [59], [60]. Since overexpressed Nab3 suppresses Htt toxicity without altering organization of Htt foci, Htt103Q-NLS appears to cause growth defects via inactivation of Nab3. In order to determine if this is the case, we carried out a structure/function analysis to determine if overexpressed Nab3 requires its essential functional domain to suppress Htt103Q-NLS toxicity. In these studies, we employed a strain (Nab3 TetR) where Nab3 expression halts when the cells are grown in the presence of doxycycline (Figure S2B). A serine to alanine point mutation within the RNA recognition motif at S399, but not at the neighboring S397, inactivates Nab3 function rendering the yeast inviable if this is the sole copy of Nab3 (Figure 3A) [60]. Nab3(S397A) is expressed at a lower level than Nab3(S399A) yet still supports normal growth. This observation suggests that Nab3(S399A) has not lost ability to complement Nab3 depletion due to low expression levels. The C-terminal polyQ-rich region of Nab3 is also required to support cell viability as expression of a version of Nab3 lacking this domain also failed to complement the deletion strain (Figure 3A).

Utilizing these Nab3 constructs to determine what region of Nab3 is required to alleviate the Htt growth defect, we examined which forms of Nab3 altered Htt103Q-NLS toxicity. The only forms of Nab3 that suppressed Htt103Q-NLS toxicity when over-expressed were the fully functional forms that were able to complement growth defects caused by Nab3 depletion (Figure 3B). Neither the polyQ-rich deletion nor the E399A forms of Nab3 alleviated the Htt103Q-NLS growth defect (Figure 3B). A non-functional Nab3 fragment that contains only its polyQ-rich containing was also insufficient to suppress toxicity.

In monitoring the potential for Nab3 to have an impact upon spatial quality control of Htt103Q-NLS, we found that a monomeric RFP (mRFP) tagged form of Nab3 and Htt103Q-NLS both localize in the nucleus (Figure 3C). At this level of expression, the Htt103Q-NLS protein forms small punctae in approximately 35% of cells expressing the construct while the remaining population exhibits a diffuse nuclear pattern [17]. When Htt103Q-NLS was co-expressed with Nab3-mRFP, the nuclear Nab3-mRFP became enriched in the location where the Htt103Q-NLS punctae formed, but the number of cells exhibiting the nuclear Htt punctae was unchanged (Figure 3C). These data suggest that Htt103Q-NLS interacts with Nab3, but Nab3 overexpression does not alter Htt103Q-NLS aggregation. In support of this hypothesis, Nab3 was also identified as an interaction partner of Htt103Q via mass spectrometry [61]. Nab3-mRFP does not co-localize with a cytoplasmic form of Htt103Q (Figure 3D), but it suppresses toxicity of Htt103Q that does not contain an NLS (Figure 3E). Htt103Q partitions between the cytosol and nucleus, so elevation of Nab3-mRFP appears to restore function of Nab3 in the nucleus without impacting spatial organization of cytosolic or nuclear Htt103Q.

Htt103Q-NLS appears to interact with Nab3, titrating it away from its normal and essential function, and suppression of toxicity by Nab3 overexpression occurs by replacing the lost functional protein. This interaction may occur through contact formation between Htt103Q-NLS and the polyQ domain of Nab3. Since the polyQ domain of Nab3 is required for Nab3 to support cell viability, such interactions appear to contribute to the cytoxicity caused by accumulation of Htt103Q-NLS in the nucleus. The Nrd1 complex is now implicated as a target involved in RNA and DNA metabolism that is inactivated by polyQ proteins that accumulate in the nucleus [29], [62].

### The PolyQ-rich Domain of Cbk1 Suppresses Proteotoxicity by Modulating the Spatial Organization of Htt103Q Foci

**Figure 4:**
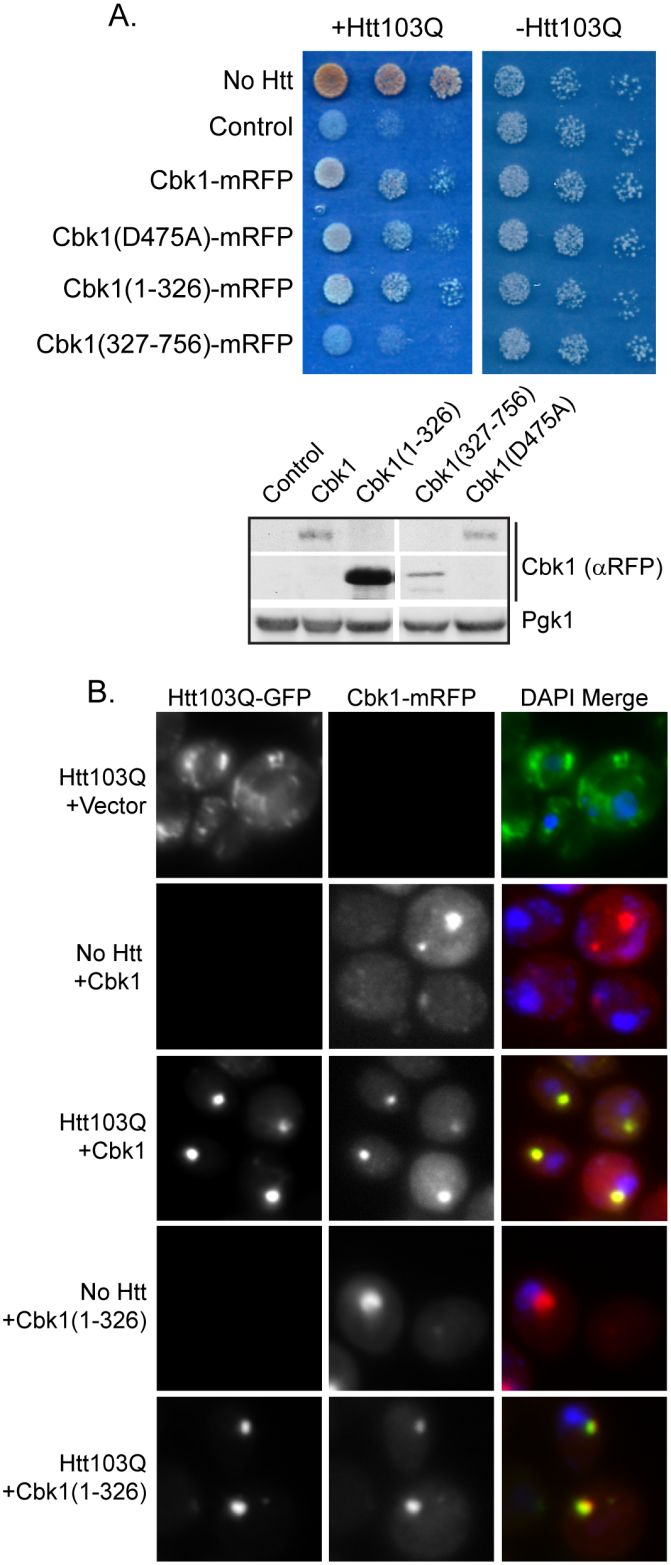
The polyQ-rich region of Cbk1 alters Htt103Q toxicity and aggregation. (A) Impact of indicated Cbk1 truncations or mutations upon Htt103Q toxicity as monitored via growth assays plated in 5-fold dilutions. Cbk1(1–326) is the polyQ-rich region of the protein, and Cbk1(327–756) lacks this region. (B) Cbk1-mRFP and Cbk1(1–326)-mRFP co-localize with Htt103Q in distinct foci. Cbk1 constructs were expressed from a copper inducible promoter induced with 100 uM CuSO_4_ for 5 h total and Htt103Q was induced with 2% galactose for 4 h total. Nuclei were visualized with DAPI staining.

Cbk1 and Pop2 suppress polyQ expanded Htt toxicity via a mechanism associated with their ability to influence the organization of Htt103Q-NLS and Htt103Q foci. Cbk1 and Pop2 are cytosolic proteins, so we carried out studies with Htt103Q to determine how they suppress polyQ toxicity. Cbk1 is a kinase of the Ndr/Lats family and functions in the regulation of Ace2 and cell morphogenesis [63]. However, this function of Cbk1 is not necessary for it to act as a Htt103Q toxicity suppressor, because over-expression of either a kinase dead form (Cbk1(D475A)-mRFP) [63] or the N-terminal portion of Cbk1 containing the polyQ-rich region, which lacks the functional domain (Cbk1(1–326)-mRFP), could still alleviate Htt103Q toxicity (Figure 4A). Conversely, expression of Cbk1 lacking the polyQ-rich region (Cbk1(327–756)-mRFP) was unable to alleviate Htt103Q toxicity even though the expression level of Cbk1(327–756)-mRFP was approximately equivalent to that of full length Cbk1 (Figure 4A). Each of the Cbk1-mRFP constructs was expressed from the Cbk1 endogenous promoter which was not titratable. Although expression levels for the Cbk1(1–326) construct are higher than the others, we find similar toxicity and microscopy results for both this construct and the full length gene. Therefore, the polyQ-rich region of Cbk1 is necessary and sufficient for Htt103Q toxicity suppression. It is notable that levels of Cbk1 required to suppress Htt toxicity are relatively low, so it could act catalytically, but kinase dead Cbk1 mutants are still active as suppressors.

As the N-terminus of Cbk1 was necessary and sufficient for toxicity suppression, we next asked if over-expression of this polyQ-rich region could also alter Htt103Q aggregation. These experiments were carried out in conjunction with the full length Cbk1 protein to ensure that the Cbk1 polyQ-rich domain exhibited similar behaviors. Interestingly, both full length Cbk1-mRFP and Cbk1(1–326)-mRFP localized to a perinuclear foci when expressed in the absence of Htt103Q (Figure 4B). This was reminiscent of Rnq1 foci when Rnq1 is highly over-expressed, however, elevation of Rnq1 causes a growth defect [13] whereas Cbk1 over-expression does not (Figure 1A). When Cbk1 or the Cbk1 polyQ-rich domain was expressed along with Htt103Q, the Htt103Q shifted from amorphous looking foci speckled throughout the cytosol, to co-localize at the perinuclear foci with Cbk1 (Figure 4B). These data suggest that the polyQ-rich region of Cbk1 interacts with Htt103Q, which mediates cytoprotective accumulation of Htt103Q in distinct foci.

### The PolyQ Domain of Pop2 Requires an Adjacent Proline-rich Motif to Modulate Spatial Quality Control and Suppress Htt103Q Toxicity

**Figure 5:**
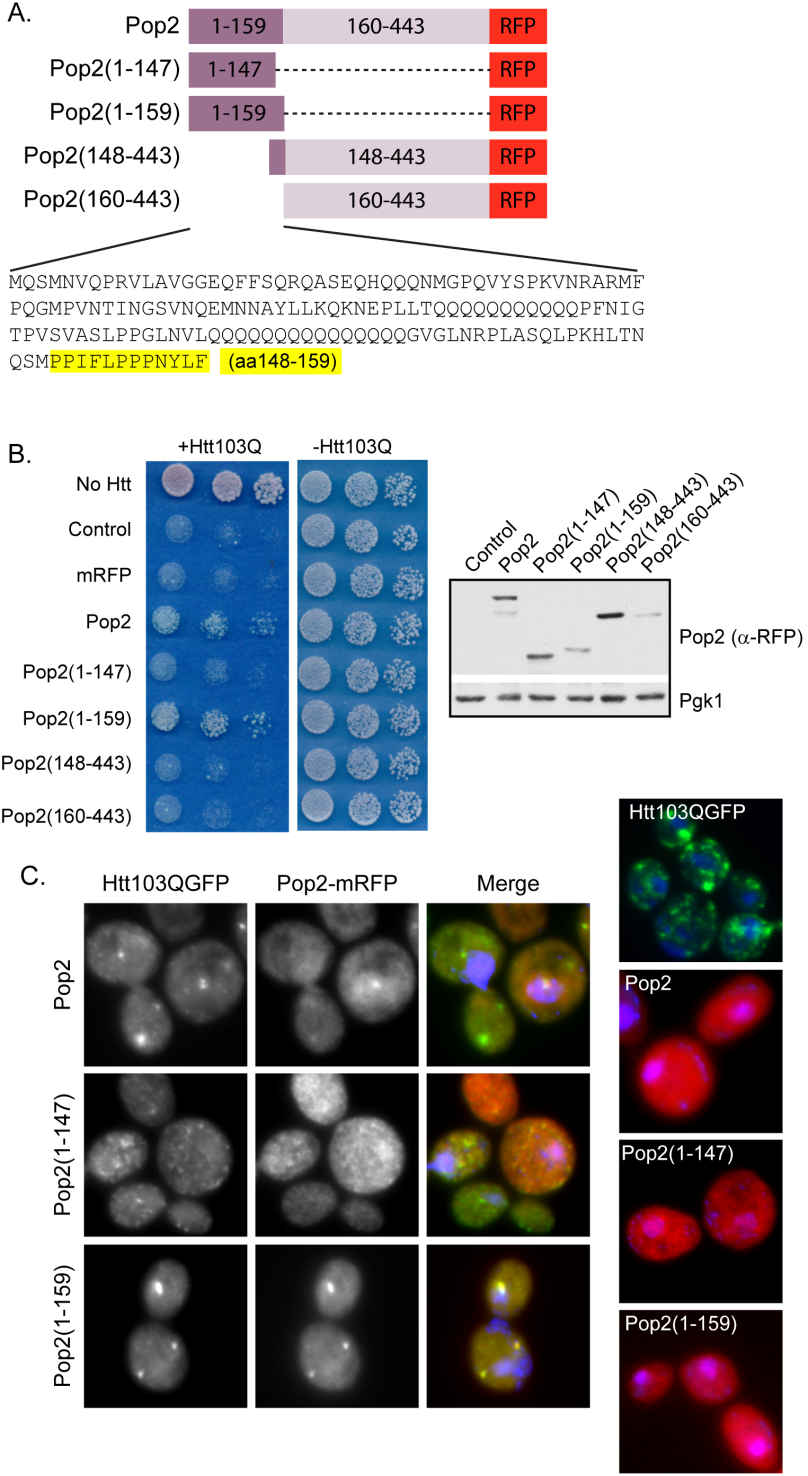
A short proline-rich stretch in the Pop2 polyQ domain is required to impact Htt103Q toxicity and aggregation. (A) A diagram illustrating the constructs utilized in these experiments and the sequence of the polyQ domain. The proline-rich region of the Pop2 polyQ domain is highlighted. Numbers indicate amino acid residues. (B) Only full length Pop2 and Pop2(1–159) suppress Htt103Q toxicity. Expression of Pop2 constructs was monitored by Western blot detection using an anti-RFP antibody. (C) Pop2 lacking the proline-rich region is unable to alter Htt103Q aggregation as monitored by fluorescence microscopy. Nuclei were visualized with DAPI staining.

Pop2, a subunit of the Ccr4/Not1 complex that functions in RNA decay [64], suppresses Htt toxicity by altering Htt103Q aggregation and Pop2 is not an essential protein. Structure/function analysis (Figure 5A) revealed that the N-terminal polyQ-rich region of Pop2(Pop2(1–159)) is necessary to suppress Htt103Q toxicity (Figure 5B). The 12 amino acids at the C-terminus of the Pop2 polyQ domain are enriched in proline residues (Figure 5A). Since the poly-proline region on Htt103Q-Pro renders it benign through targeting it for protective aggregation [35], [36], we asked whether the proline rich region at the end of Pop2(1–159) acts in similar manner. Excitingly, this indeed appears to be the case as expression of Pop2(1–147) was unable to suppress Htt103Q toxicity (Figure 5B). Proline-rich residues 148–159 of Pop2 were not sufficient to alleviate toxicity without the polyQ-rich region, however, as expression of Pop2(148–443) was unable to suppress Htt103Q toxicity (Figure 5B).

In order to determine if there was a correlation between toxicity suppression and a change in Htt103Q aggregation, we monitored the microscopic aggregation pattern of Htt103Q during co-expression with Pop2. Expression of full length Pop2 or Pop2(1–159) caused Htt103Q to shift from the amorphous looking speckled punctae throughout the cytosol to distinct perinuclear foci (Figure 5C). Pop2(1–147) which lacked the short proline-rich region, however, was unable to alter Htt103Q aggregation (Figure 5C). Unlike over-expressed Cbk1, which formed perinuclear foci, each of the Pop2 constructs was expressed in a diffuse pattern throughout the cell (Figure 5C) indicating that Pop2 and Cbk1 seem to suppress Htt103Q toxicity in different ways. Upon expression of Htt103Q, however, Pop2 and Pop2(1–159) co-localized with Htt103Q in the perinuclear foci (Figure 5C) suggesting an interaction between these proteins results in their co-localization in foci. When expressed together, Htt103Q and Pop2(1–147) did not co-localize indicating that the proline-rich region following the polyQ domain of Pop2 is required for the putative interaction.

Using gel filtration chromatography, we verified that Pop2(1–159) indeed was found in complex with aggregated Htt103Q (Figure S3A). Pop2(1–159) migrated as a monomer around the same molecular weight as the monomeric Htt103Q, but a pool of Pop2(1–159) also migrated as a species close to 200 KDa (Figure S3A). When Pop2(1–159) and Htt103Q were co-expressed, the 200 KDa species of Pop2 shifted to the high molecular weight complex with Htt103Q (Figure S3A). In agreement with co-localization studies, the Pop2(1–147) construct lacking the proline-rich region did not co-migrate with the high molecular weight Htt103Q (Figure S3A). Additionally, Htt103Q and Pop2(1–159) co-precipitated from the high molecular weight fractions, but not the monomeric fractions providing evidence of an interaction or complex containing these proteins (Figure S3B).

In contrast to Nab3, the polyQ-rich domains of Cbk1 and Pop2, rather than the functional domains, alter Htt103Q toxicity and this action correlates with changes in the spatial organization of Htt103Q. These data suggest that interaction of Htt103Q with certain polyQ domains is sufficient to change the cells ability to package Htt103Q into benign species. These results were obtained during overexpression of polyQ-rich domains, but Cbk1 and Pop2 are not extremely abundant proteins. Thus, whether or not the endogenous forms of these proteins have a role in polyQ toxicity remains unclear. Yet, cells express a large number of polyQ domain proteins [65], so these data provide an example of how the collective action of proteins with polyQ-rich protein domains could impact the spatial organization of aggregated polyQ proteins.

### Pop2 and Cbk1 Alter Htt103Q Toxicity and Localization in a Sti1-dependent Manner

**Figure 6:**
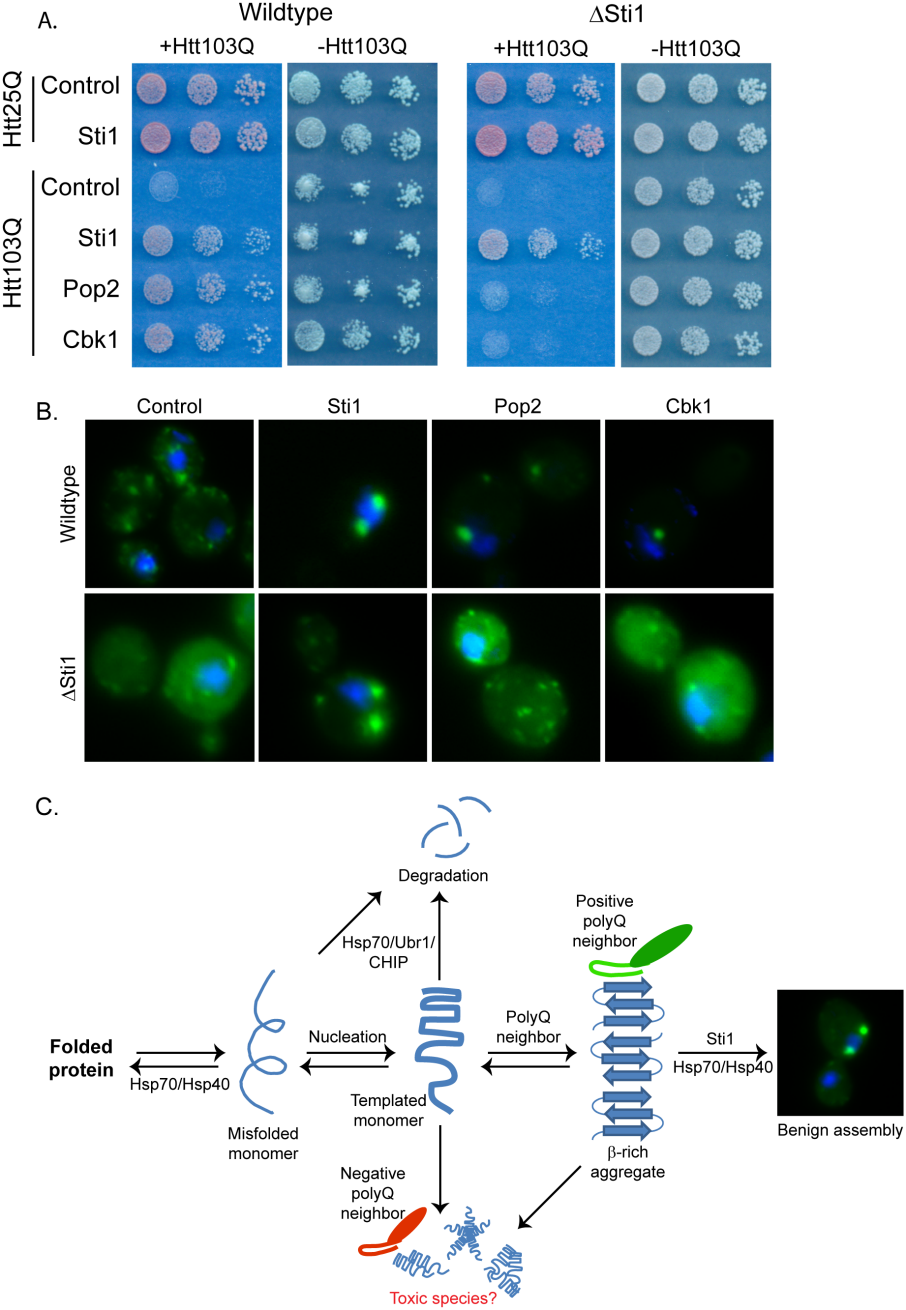
Cbk1 and Pop2 act in a Sti1-dependent manner. (A) Impact of Sti1 deletion upon Pop2 and Cbk1 ability to suppress Htt103Q toxicity. Growth assays were plated in 5-fold dilutions on glucose (−Htt) or galactose (+Htt). (B) Impact of Sti1 deletion upon Pop2 and Cbk1 ability to alter Htt103Q aggregation. Fluorescence microscopy was carried out as above, and nuclei were visualized using DAPI staining. (C) Model for Htt103Q benign aggregate assembly.

Pop2 and Cbk1 alter the spatial distribution of Htt103Q in a manner similar to what is observed upon modulation of the activity of the of the Hsp70 co-chaperone Sti1 [43]. Sti1 is a Htt103Q toxicity suppressor identified in the screen that acts with Hsp70 to promote accumulation of SDS-resistant material, including Htt103Q, in a perinuclear quality control center that appears distinct from the aggresome [43]. Sti1 is required for protective sequestration of templated Htt103Q into quality control centers with potentially toxic amyloid-like material [43]. Yet, Sti1 differs from Pop2 and Cbk1, in that it can modulate the spatial organization and toxicity of Rnq1 prions as well as Htt103Q, so Sti1 appears to be generally involved in spatial organization of amyloid-like proteins [43]. Therefore, we tested whether or not Pop2 and Cbk1 acted in a manner that is dependent upon Sti1 to modulate spatial quality control and toxicity of Htt103Q. Interestingly, Pop2 and Cbk1 each lost its ability to suppress Htt103Q toxicity in a *sti1Δ* strain (Figure 6A). Pop2 and Cbk1 action in suppression of Htt toxicity is therefore Sti1 dependent. Changes in Htt103Q spatial organization caused by Pop2 and Cbk1 are also dependent upon Sti1 (Figure 6B) as they lose their ability to redistribute Htt103Q into distinct foci in the absence of Sti1. Pop2 and Cbk1 act in a Sti1 dependent spatial quality control pathway to suppress growth defects caused by Htt103Q expression in yeast (Figure 6C).

## Discussion

Data presented herein illustrate how interactions between disease proteins that contain an expanded polyQ repeat and cellular proteins that contain normal polyQ domains differentially affect protein homeostasis. Htt103Q disrupts protein metabolism through globally impacting the functions of proteins in complexes involved in DNA and RNA metabolism that contain polyQ domains [26]–[29]. We contribute to this concept via identification of Nab3 and the Nrd1 RNA processing complex as a target of toxic forms of Htt103Q. Interestingly, data obtained with the polyQ proteins Pop2 and Cbk1 also suggest that some interactions between Htt103Q and cellular polyQ domains result in the cytoprotective targeting of disease proteins to the Sti1/Hsp70 dependent spatial quality control pathway [43]. Thus, it appears that polyQ proteotoxicty can be affected by the balance of positive and negative interactions between polyQ conformers, Sti1/Hsp70, and normal polyQ-rich proteins.

Cbk1 and Pop2, when over-expressed, both drive spatial reorganization of Htt103Q via a pathway that is dependent upon the Hsp70 co-chaperone Sti1. Thus, Pop2 and Cbk1 suppress proteotoxicity by acting through a chaperone dependent spatial quality control pathway to drive accumulation of toxic Htt103Q to a benign location. Sti1 acts with Hsp70 and Sis1 in a spatial quality control pathway to orchestrate the formation of benign perinuclear foci containing amyloid-like material such as Htt103Q [43]. Interestingly, Pop2, Cbk1, and Sti1 were all discovered in the same screen, and Pop2 and Cbk1 over-expression are unable to promote protective aggregation in the absence of Sti1. These observations place the polyQ neighbors of Htt103Q upstream of Sti1 in a chaperone dependent facilitated aggregation pathway (Figure 6C).

An additional screen hit, YHR127w, may also function in Hsp70 dependent spatial quality control and facilitate protective aggregation of amyloid-like protein species. YHR127w has no known function, and is the gene within GF7 that acts independently to suppress Htt103Q toxicity. Insight into YHR127w function is minimal, yet a large scale protein:protein interaction study suggests that YHR127w is an interaction partner of Hsp70 [66]. Suppression of Htt103Q toxicity by YHR127w also correlates with a change in the spatial organization of Htt103Q. Since YHR127w and Sti1 were hits in the same high copy suppressor screen, it is possible that YHR127w functions with Sti1 and Hsp70 in spatial quality control to detoxify polyQ-rich proteins. However, the biological function of YHR127w requires further investigation.

Approximately 1–3% of eukaryotic proteomes contain proteins with a polyQ stretch of at least 5 residues [67], [68]. Some of these polyQ stretch proteins are capable of interacting with the expanded polyQ domains of disease proteins [6], [28], [45], [48], [61], [69], [70], and in most cases such interactions seem to have negative outcomes on protein function and cell viability [6], [7], [48], [69], [70]. In one instance, interaction between Htt103Q and cellular proteins appears to seed formation of toxic Htt103Q [13], [17], [34], [58], [71]. Over-expression of Pop2 and Cbk1 promotes the organization of Htt103Q into benign foci, thus, these proteins fall into a unique category of polyQ containing proteins that have a positive effect on disease related polyQ. Why there are both positive and negative outcomes of Htt interaction with polyQ protein neighbors remains unclear, yet there are several scenarios that could explain these differences.

Flanking sequences of the polyQ proteins have a profound effect upon polyQ toxicity and aggregation [35], [36], [72]. Over-expression of the polyQ domain of Pop2 can direct Htt103Q to a perinuclear foci, but only if a short proline-rich stretch following the Pop2 polyQ region is present. Interestingly, the polyQ stretch in Htt exon 1 is followed by a poly-proline stretch, and addition of the this stretch to Htt103Q (in *cis*) or to Htt25Q (in *trans*) serves to detoxify Htt103Q in yeast [35], [36], [73]. Reduced toxicity correlates with accumulation of Htt103Q at a perinuclear location that corresponds with spindle pole body markers [35], [58]. Thus, Pop2 may have the ability to act in *trans* to target Htt103Q to benign quality control centers via a mechanism involving the Pop2 proline-rich stretch. However, the aggregation of non-toxic forms of Htt103Q containing a proline stretch does not require Sti1 [43]. Since Pop2 requires Sti1 to suppress Htt103Q toxicity, an alternative possibility is that the proline-rich stretch in Pop2 is simply required for the Pop2 polyQ domain to adopt a conformation that is capable of interacting with Htt103Q. Indeed, the proline-rich stretch of Htt103Q also impacts the conformation of Htt [72], [74], [75]. Thus, the Q-rich region of Pop2(1–159) might be stabilized in an alternate conformation as compared to Pop2(1–147), which lacks the proline-rich stretch. The conformational shift may change the ability of the protein to interact with Htt103Q as Pop2(1–159) is found in perinuclear foci with Htt103Q while Pop2(1–147) is not.

Subtle differences in polyQ domains of the polyQ-rich proteins may also help determine whether there is a positive or negative proteotoxic outcome. Nab3, Cbk1, and Pop2 each contain polyQ-rich regions, but little else is similar between these proteins as seen by their alignment (Figure S1C). The polyQ domain of Nab3 contains multiple prolines and other nonpolar residues interspersed throughout the many short polyQ stretches, while these residues mainly follow 2 polyQ tracts in Pop2. Cbk1 has two longer polyQ stretches separated by a region rich in polar residues (glutamine, asparagine, and serine), and these are interspersed with hydrophobic residues. Therefore, surfaces exposed by polyQ domains that interact with Htt103Q are different, and such interactions may positively or negatively influence the conformation/function of the polyQ domain containing protein and also the conformation of Htt103Q.

Altogether, there is a complex relationship between polyQ containing proteins where Htt103Q can interfere with normal function of certain polyQ proteins, such as Nab3, while other polyQ proteins, such as Cbk1 and Pop2, can interfere with toxicity of Htt103Q. A chaperone dependent pathway for protective aggregation regulates spatial quality control of amyloid-like proteotoxic proteins. Variations in the relative abundance of chaperones in this facilitated aggregation pathway as well as components within a polyQ interaction network may contribute to maintenance of normal protein function and cellular viability during toxic insult. Alteration of levels of certain PQC components as cells age contributes to a decline in protein homeostasis leading to various diseases such as in neurodegenerative disorders [76]. Thus, changes in levels of components within the spatial quality control pathway described here may occur in a certain cell populations over time as in aging. Differences in relative abundance of these components may also occur from cell type to cell type contributing to differential susceptibility of specific neuronal populations in polyQ disorders. Further studies will better define this complex interaction network and how it impacts mechanisms for protective packaging and aggregation of polyQ disease proteins.

## Materials and Methods

### Strains and Plasmids

Yeast strains and plasmids used in this study are listed in Tables S1 and S2. All strains harbored Rnq1 in its [*RNQ^+^*] prion form unless otherwise indicated. The generation of isogenic [*rnq−*] strains was accomplished via sequential passage of cells on plates containing 3 mM guanidinium-HCl. Plasmid transformation into yeast was performed using a lithium acetate method as described previously [13]. Yeast cell culture was carried out in synthetic media containing the appropriate amino acids for selection of auxotrophic markers. The Htt25Q and Htt103Q (both with and without the NLS) integration strains were generated as described previously [43]. The Htt construct used in these studies consisted of the first 17 amino acids of Htt exon 1 followed by 25 or 103 glutamine residues, and lacked the proline-rich region. It also contained an N-terminal FLAG tag and a C-terminal GFP tag. During microscopy and Western blot experiments, expression of Htt was induced from the galactose promoter with 2% galactose for 4 h. Cbk1-mRFP constructs and Nab3-mRFP were all expressed from a CUP1 promoter induced with 100 uM CuSO_4_ for 5 h. Each of the remaining suppressor constructs were expressed constitutively from their endogenous promoters. Rnq1-GFP was also expressed from a CUP1 promoter but no additional copper was added to the media in order to induce low level overexpression without causing toxicity, yet be able to observe Rnq1 by fluorescence microscopy.

### High Copy Screen for Toxicity Suppressors

The Htt103Q-NLS integration strain was transformed with a 2 µ yeast expression library [77]. Plasmids in this library carried a 5–10 Kb fragment of genomic DNA. Transformations were plated on synthetic media containing 2% galactose to induce expression of the toxic Htt103Q-NLS construct. Colonies which were able to grow under these conditions after 3 days on solid media were isolated and screened using 5-FOA. This step was to ensure that isolates were not able to grow under toxic conditions due to a mutation and growth on galactose media was plasmid dependent. A plasmid rescue experiments was carried out on each colony which exhibited plasmid dependent growth. These plasmids underwent a sequencing reaction to determine the 5′ and 3′ ends of the DNA fragment which was able to suppress Htt103Q-NLS toxicity. Numerous gene regions (which were not only the same DNA fragment, but had overlapping DNA regions) were found multiple times suggesting the screen neared saturation. ORFS in each suppressor were subcloned into a pRS426 plasmid along with approximately 400 bp 5′ and 3′ UTR regions so that the protein would be expressed from its endogenous promoter to similar levels as those during the original screen.

### Toxicity Assays

Yeast samples were normalized to the same OD_600_, then 5 fold serial diluted with sterile distilled water in a 96-well plate. Dilutions were plated in 10 uL spots on appropriate synthetic dropout media. Most images of yeast grown on glucose were scanned at 48 h and galactose at 72 h. Htt103Q toxicity was assessed on plates containing 2% galactose, and in the case of Cbk1-mRFP constructs, supplemented with 500 uM CuSO_4_. Control growth plates contained 2% glucose.

### Fluorescence Microscopy

Fluorescence microscopy was carried out as described previously [17]. Briefly, cultures were fixed with 4% formaldehyde and 0.1 M KPO_4_ pH 7.4, and stored in 0.1 M KPO_4 _pH 7.4 with 1.2 M sorbitol. Cells were then permeabilized with 0.1% Triton and stained with 0.5 ug/mL DAPI. An Olympus IX81 motorized inverted fluorescence microscope paired with Metamorph software was used to collect images. Micrographs were merged and processed using ImageJ (NIH) and Photoshop (Adobe). In order to be unbiased, counting and quantitation of foci was performed using the thresholding feature of ImageJ.

### Nab3 Depletion

The Nab3-TetR strain shuts down expression of Nab3 upon addition of doxycycline to the growth medium. This strain was transformed with the indicated plasmids, and colonies were expanded in liquid culture. At mid-log phase growth, 10 ug/mL doxycycline was added to the media. Samples were taken at the indicated timepoints starting at time of drug addition (T = 0) out to 20 h post doxycycline. Samples were lysed and run on SDS-PAGE followed by Western blotting for endogenous Nab3.

### Western Blotting

Lysates were prepared from pellets of yeast by either mechanical disruption via bead lysis (for SDD-AGE, co-IP, and gel filtration) as described previously [13], [17] or an alkali lysis method as described previously [42]. When using mechanical disruption, the lysis buffer utilized was 0.1% Triton X-100, 75 mM Tris pH 7.4, 150 mM NaCl, 1 mM EDTA, 1 mM PMSF, and Sigma protease inhibitor cocktail. Standard separation techniques were employed to analyze lysates via SDS-PAGE. Protein was transferred to nitrocellulose for 75–90 min at 110 V, and probed using the indicated antibodies. Antibodies used in this study are listed in Table S3.

### SDD-AGE

Semi-denaturing detergent agarose gel electrophoresis (SDD-AGE) was carried out as described previously [13]. Briefly, Htt103Q was induced for 4 h in cells expressing either empty vector, or the indicated toxicity suppressor under control of their respective endogenous promoters. Samples were collected, lysed, and normalized (DC protein assay kit; Bio-Rad) and equivalent amounts of total protein were loaded in tandem on a standard SDS-PAGE gel (to monitor total levels of Htt103Q) and in a 1.4% agarose gel containing 0.1% SDS (to monitor SDS-resistant Htt103Q). The agarose gel was run at 90–100 V for approximately 2 h, then transferred to PVDF at 12 V for 15 h and Western blotting was carried out.

### Gel Filtration Chromatography and Co-immunoprecipitation

Cell lysates were prepared via mechanical disruption using the lysis buffer listed above. A 500 uL loop was loaded with lysates at a concentration of 7 mg/mL total protein which was then injected through a sephacryl S-200 gel filtration column (GE Healthcare) at a rate of 0.25 mL/min. Fractions of 1 mL were collected and run on a 10% SDS-PAGE gel, followed by standard Western blotting. For co-IP from column fractions, a 400 uL aliquot of the indicated fractions were incubated with anti-GFP (to bind the Htt103Q) followed by incubation with a 50/50 Protein G bead slurry. After the beads were washed with lysis buffer, they were resuspended in sample buffer and boiled for 10 min prior to loading on a 10% SDS-PAGE gel, followed by Western blotting. Pop2(1–159)-mRFP was detected using polyclonal anti-RFP antibody and Htt103Q was detected using polyclonal anti-YFP (C. Beckers).

## Supporting Information

Figure S1 **PolyQ-rich protein domain details.** (A) Diagram of polyQ-rich proteins. Numbers indicate amino acid residues. (B) Sequences of polyQ-rich regions in Nab3, Pop2, and Cbk1. (C) ClustalW2 alignment of sequences from (B). Color legend is indicated.
(TIF)

Figure S2 **Prion status of Rnq1 is unaffected by Pop2 and Nab3 is depleted by doxycycline.** (A) Pop2 does not alter Rnq1 localization or prion status. [RNQ+] prion status was monitored via aggregation of Rnq1-GFP expressed from CUP1 promoter at basal levels of copper in media. (B) Doxycycline treatment inhibits expression of Nab3 as monitored by Western blot.
(TIF)

Figure S3 **Pop2(1–159) is found in complex with Htt103Q.** (A) Impact of Pop2(1–159)-mRFP upon Htt103Q aggregation as monitored by size exclusion chromatography. Samples were prepared as indicated in methods from cultures expressing Htt103Q alone, Pop2(1–159)-mRFP alone, or both Htt103Q and Pop2(1–159)-mRFP, as well as Pop2(1–147) alone or in conjunction with Htt103Q. (B) Interaction of Pop2(1–159)-mRFP with high molecular weight Htt103Q as monitored by co-IP. Htt103Q was precipitated from column fractions indicated.
(TIF)

Table S1 Yeast strains used in study.
(XLSX)

Table S2 Plasmids used in study.
(XLSX)

Table S3 Antibodies used in study.
(XLSX)
