# Supplementary figures and images for: Polyglutamine-Rich Suppressors of Huntingtin Toxicity Act Upstream of Hsp70 and Sti1 in Spatial Quality Control of Amyloid-Like Proteins
Source: PLoS One. 2014 May 14;9(5):e95914. doi: 10.1371/journal.pone.0095914 (PMC4020751; doi:10.1371/journal.pone.0095914)

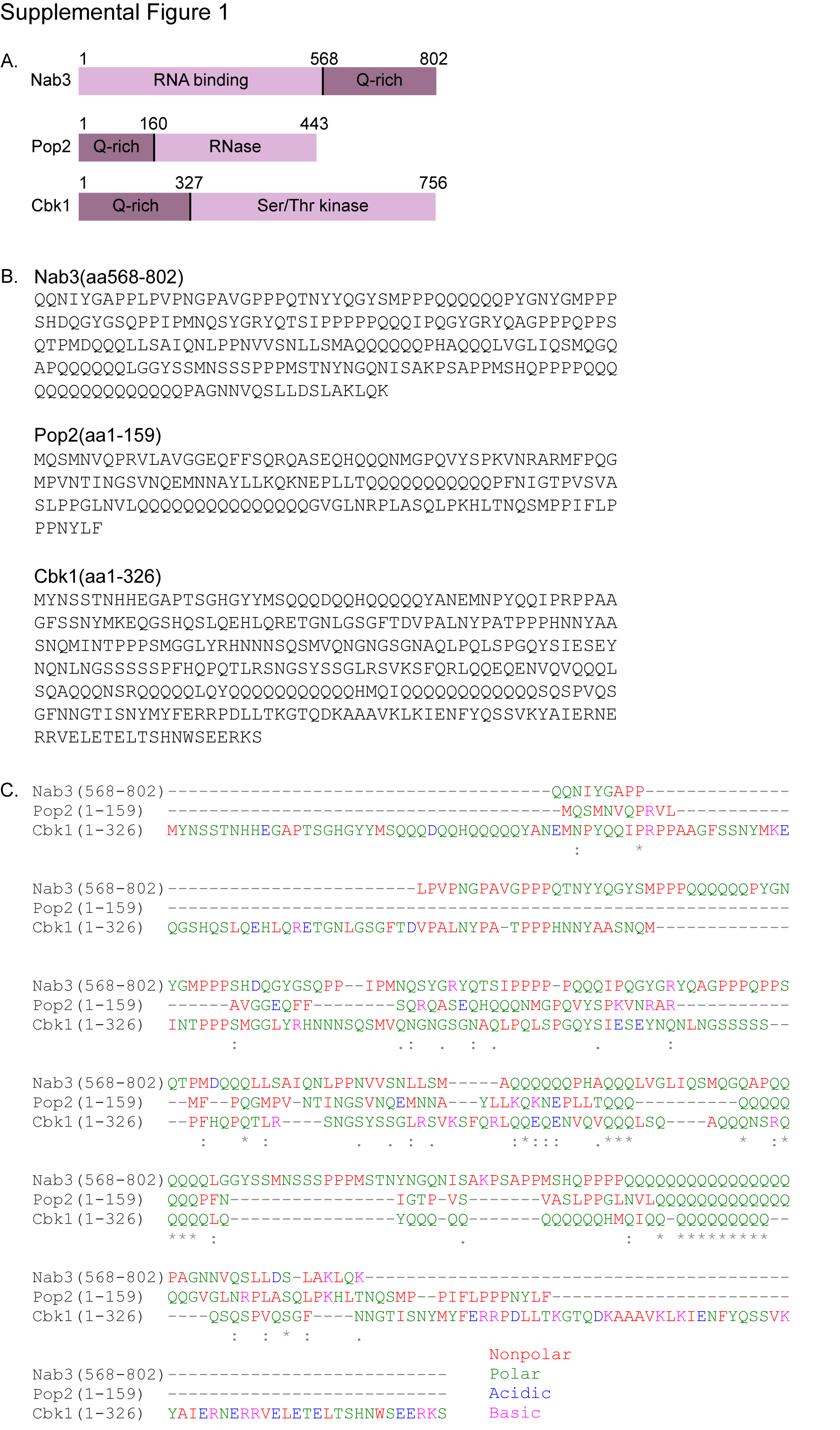

Supplement: Figure S1 — PolyQ-rich protein domain details. (A) Diagram of polyQ-rich proteins. Numbers indicate amino acid residues. (B) Sequences of polyQ-rich regions in Nab3, Pop2, and Cbk1. (C) ClustalW2 alignment of sequences from (B). Color legend is indicated. (TIF) [file pone.0095914.s001.tif]

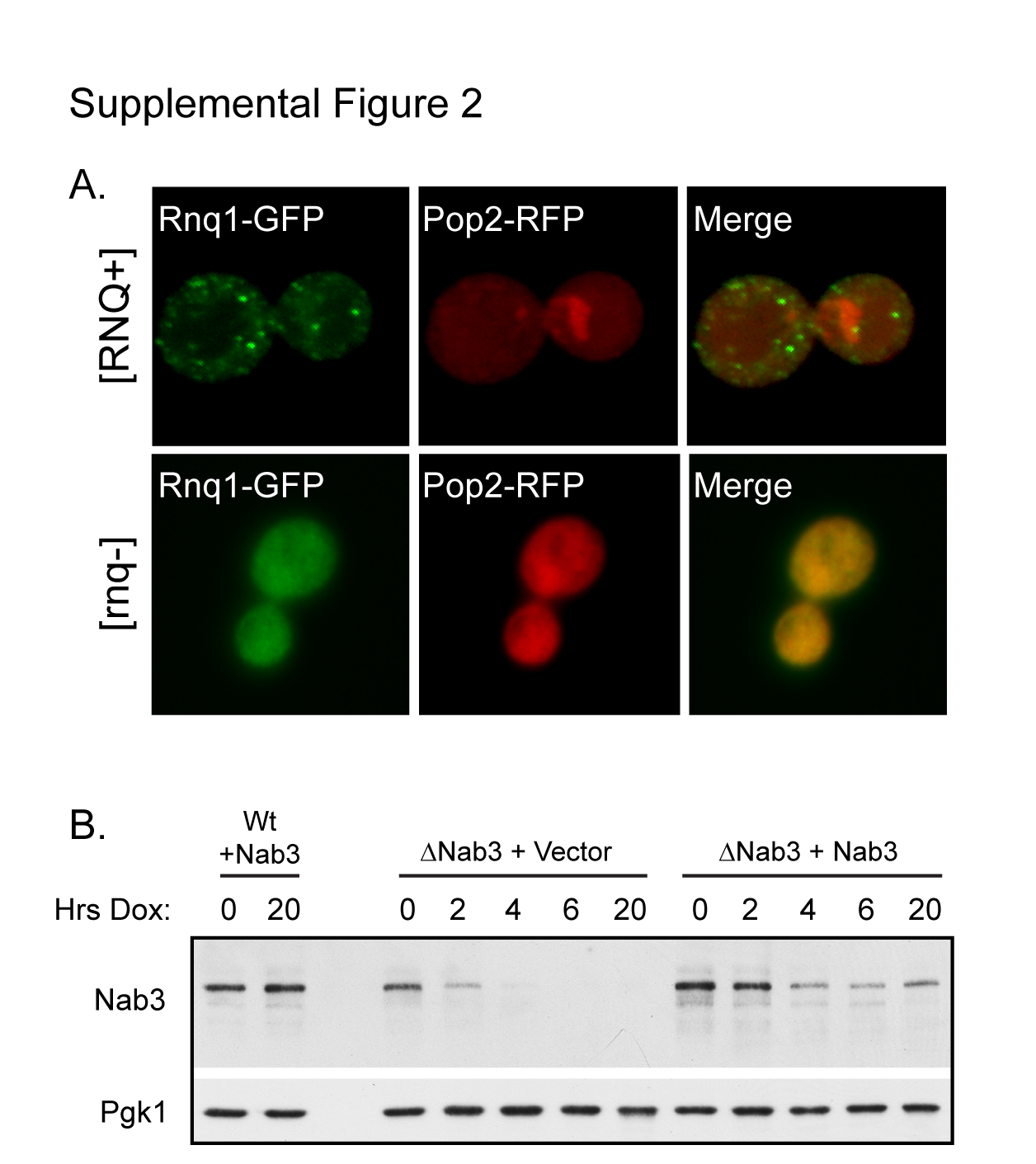

Supplement: Figure S2 — Prion status of Rnq1 is unaffected by Pop2 and Nab3 is depleted by doxycycline. (A) Pop2 does not alter Rnq1 localization or prion status. [RNQ+] prion status was monitored via aggregation of Rnq1-GFP expressed from CUP1 promoter at basal levels of copper in media. (B) Doxycycline treatment inhibits expression of Nab3 as monitored by Western blot. (TIF) [file pone.0095914.s002.tif]

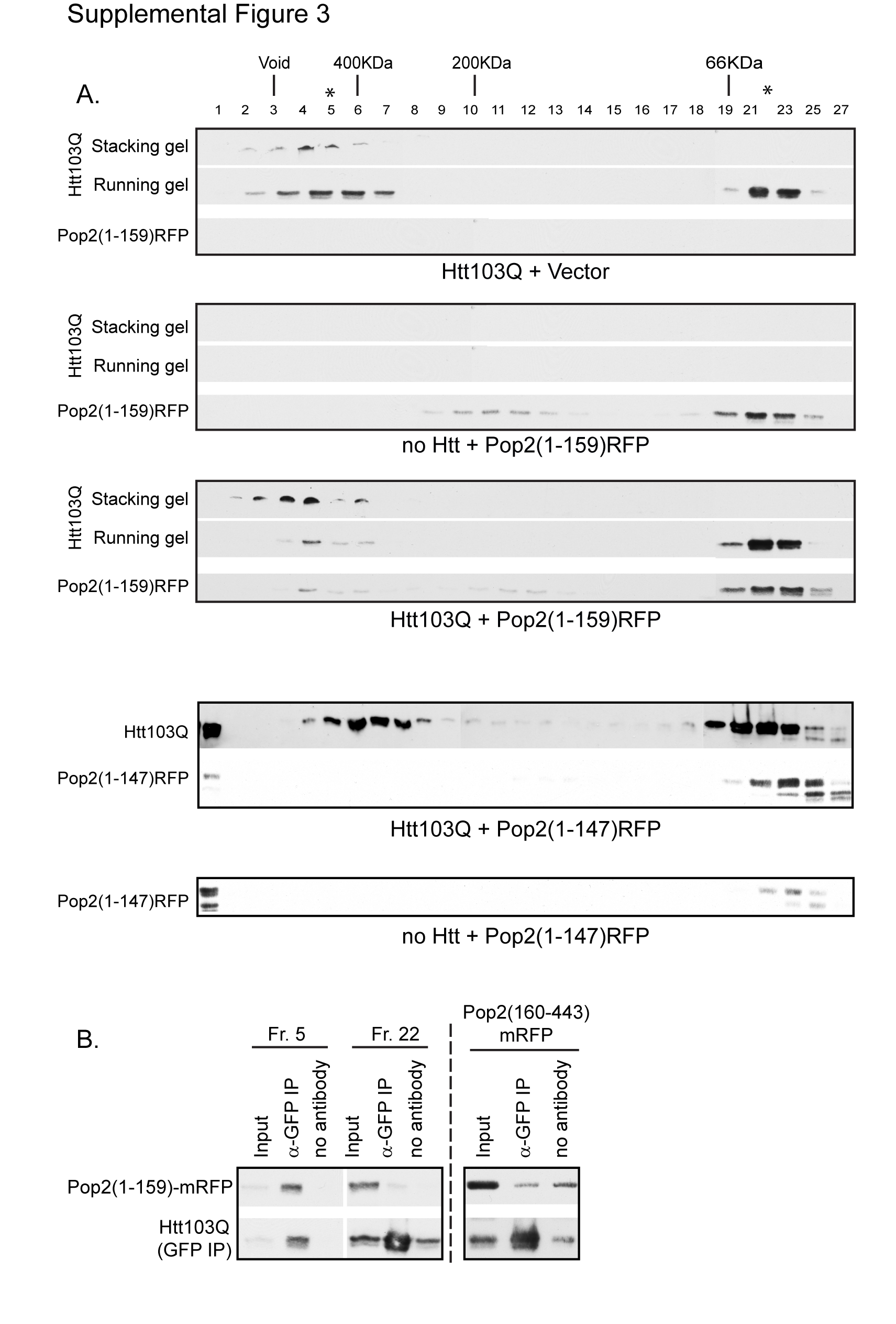

Supplement: Figure S3 — Pop2(1–159) is found in complex with Htt103Q. (A) Impact of Pop2(1–159)-mRFP upon Htt103Q aggregation as monitored by size exclusion chromatography. Samples were prepared as indicated in methods from cultures expressing Htt103Q alone, Pop2(1–159)-mRFP alone, or both Htt103Q and Pop2(1–159)-mRFP, as well as Pop2(1–147) alone or in conjunction with Htt103Q. (B) Interaction of Pop2(1–159)-mRFP with high molecular weight Htt103Q as monitored by co-IP. Htt103Q was precipitated from column fractions indicated. (TIF) [file pone.0095914.s003.tif]
